# Use of plant growth-promoting bacteria to facilitate phytoremediation

**DOI:** 10.3934/microbiol.2024021

**Published:** 2024-06-12

**Authors:** Elisa Gamalero, Bernard R. Glick

**Affiliations:** 1 Dipartimento di Scienze e Innovazione Tecnologica, Università del Piemonte Orientale, Viale T. Michel 11, Alessandria, 15121, Italy; 2 Department of Biology, University of Waterloo, Waterloo, ON, Canada N2L 3G1

**Keywords:** phytoremediation, environmental contamination, plant growth-promoting bacteria, soil bacteria, organic contaminants, metal contaminants

## Abstract

Here, phytoremediation studies of toxic metal and organic compounds using plants augmented with plant growth-promoting bacteria, published in the past few years, were summarized and reviewed. These studies complemented and extended the many earlier studies in this area of research. The studies summarized here employed a wide range of non-agricultural plants including various grasses indigenous to regions of the world. The plant growth-promoting bacteria used a range of different known mechanisms to promote plant growth in the presence of metallic and/or organic toxicants and thereby improve the phytoremediation ability of most plants. Both rhizosphere and endophyte PGPB strains have been found to be effective within various phytoremediation schemes. Consortia consisting of several PGPB were often more effective than individual PGPB in assisting phytoremediation in the presence of metallic and/or organic environmental contaminants.

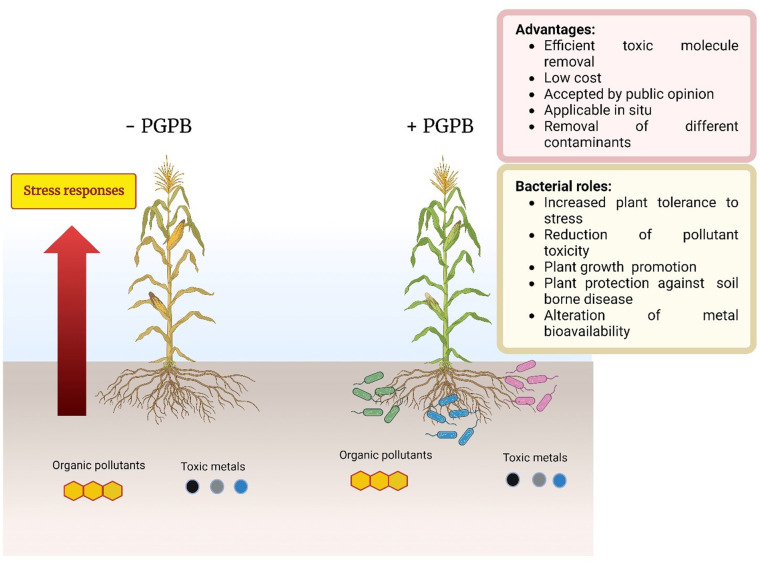

## 1. Introduction

It has been estimated that “pollution was responsible for 9 million premature deaths in 2015–2019 making it the world's largest premature risk factor of disease and premature death” [1]. In addition, more than 90% of the pollution-related deaths occurred in middle- and low-income countries including India and China. Since that time, if anything, things have gotten worse. Today, human-caused climate change accompanied by increasing levels of pollution on a global scale are no longer subjects for debate as the world becomes increasingly polluted. Moreover, as the world's population, which is ~8 billion people (worldpopulationreview.com/; accessed June 2, 2024), continues to increase and is estimated to reach ~10 billion by 2050, and as we continue to pollute our environment, toxic metals and organic compounds continue to accumulate on planet Earth and within plants, animals and human beings (www.cdc.gov/biomonitoring/environmental_chemicals.html; accessed June 2, 2024). For example, it was reported that, in many sparsely populated areas of the globe which are themselves not a source of environmental toxicants, such as the world's coral reefs, many of these regions are contaminated with copious amounts of plastic debris [2]. This marine plastic pollution is in addition to the widespread presence of plastic debris found globally in lakes, rivers, and reservoirs [3]. In addition to global plastic pollution, there are many thousands of toxic waste sites in the majority of the countries of the world. These sites may contain metals such as “lead, zinc, cadmium, selenium, chromium, cobalt, copper, nickel, or mercury”; inorganic compounds such as “arsenic, sodium nitrate, ammonia or phosphate”; radioactive compounds such as “uranium, cesium or strontium”; or organic compounds such as solvents, explosives, petroleum hydrocarbons, pesticides, and polycyclic aromatic hydrocarbons [4].

Traditionally, the cleanup or remediation of metal-contaminated soils generally includes the physical removal of the contaminated soils to secured landfill sites. This approach also requires that subsequently to the metal removal, the contaminated soil site must be restored to a natural state; in total this approach is quite expensive resulting in only a small number of the tens of thousands of contaminated sites being remediated. On the other hand, it is technically less difficult to remove many organic compounds/contaminants (compared to metals) from the environment since they can often be metabolized (remediated) *in situ* by various bacteria, some of which may already be present in the contaminated soil and others which can be added to the soil [5]. However, the breakdown *in situ* of organic compounds in soil is generally a slow and inefficient process.

**Figure 1.**
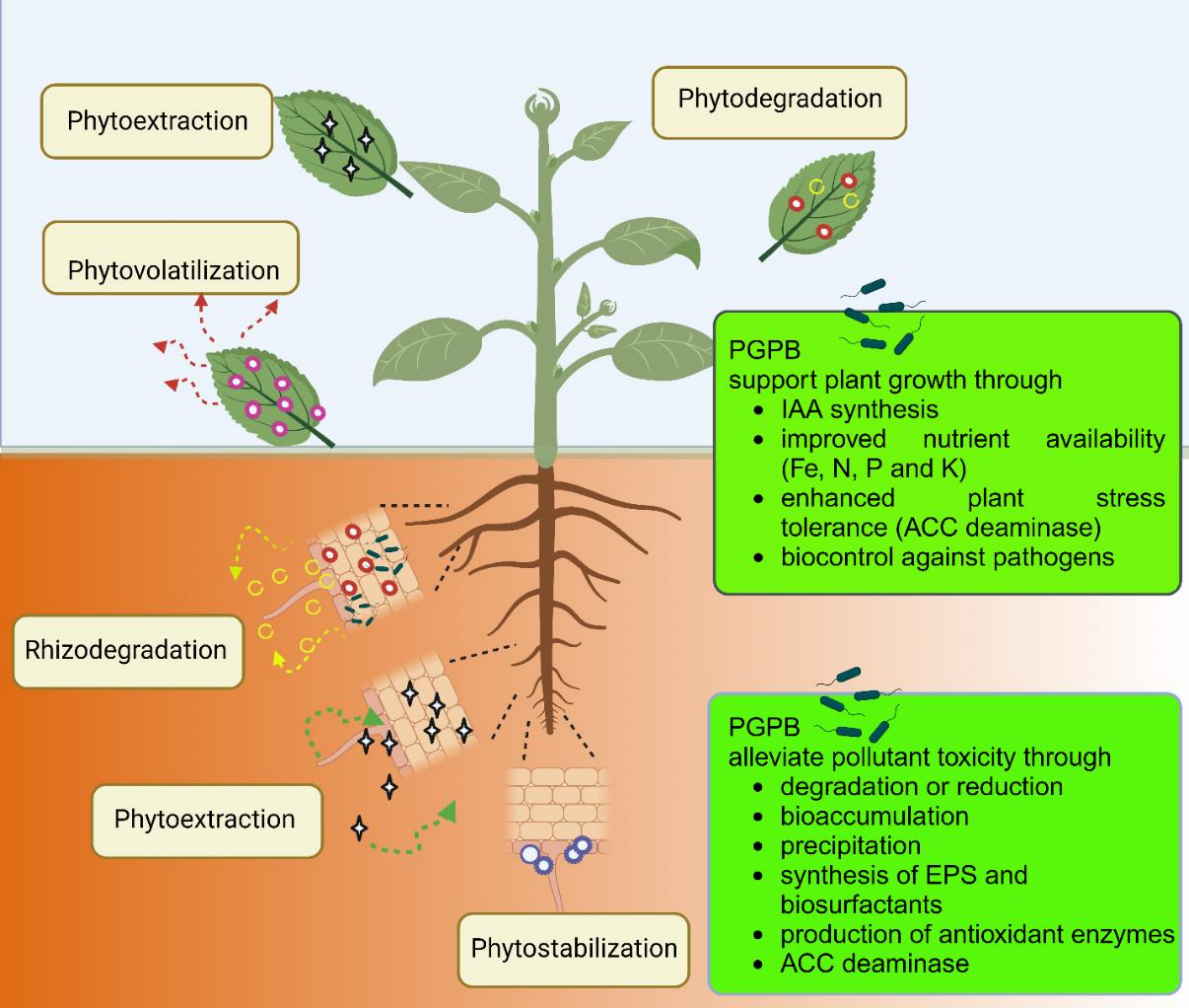
Comprehensive overview of the major mechanisms of phytoremediation and of the way in which PGPB colonizing plants can assist phytoremediation of both inorganic and organic pollutants. Phytostabilization involves the use of plants able to absorb or precipitate the pollutant (blue circles) through immobilization of the molecules in the rhizosphere thus reducing the bioavailability of the contaminant and preventing its diffusion into ground water. Phytoextraction is based on the plant's ability to absorb contaminants (black stars) at the root level and translocate them to the shoots. This mechanism is mainly used for restoring soils from heavy metal pollution and has the advantage of recovering high amounts of metals from leaf and stem tissues. Phytodegradation is mainly used in the remediation of organic pollutants and takes advantage of the capability of the plant to metabolize or detoxify the contaminant (red circles) by the synthesis of specific enzymes thereby producing compounds that are less toxic (yellow open circles). Phytovolatilization is based on the ability of a plant to take up pollutant molecules at the root level, transfer them to the leaves and transform them into volatile compounds which are then released into the environment (purple circles). This tool is often exploited in mercury and arsenic remediation. Rhizodegradation (or Phytostimulation) consists of the degradation of organic pollutants by the microorganisms living in the rhizosphere or inside the plant tissues (endophytes). This is a cooperative degradation process where the plants support microbial survival through root exudation and microorganisms living on and in the root metabolize the pollutant (red closed circles) and release the product into the soil (yellow open circles). Here, PGPB support plant growth by direct (auxin synthesis, siderophores, improvement of nutrient availability) and indirect (biocontrol) mechanisms. PGPB can also favor plant development in a polluted site by relieving contaminant toxicity. The main mechanisms used by PGPB are the synthesis of ACC deaminase (and the reduction of the stress ethylene level), bioaccumulation and bioprecipitation (lowering the pollutant availability), direct catabolism on the contaminant, production of EPS and biofilm formation (EPS can interact with pollutant molecules via several mechanisms leading to increased bioavailability and easier and faster enzymatic breakdown) and release of antioxidant enzymes (protecting the plant against pollutant toxicity). This figure has been created using BioRender™.

Given the highly inefficient and expensive methods that have been available for removing toxic metals and/or organic compounds from the environment, a little more than 30 years ago scientists began developing the process of phytoremediation, a clean, effective, and relatively inexpensive technology to perform this task [6]. Phytoremediation is generally defined as the removal or stabilization of toxic substances from the environment by plants [7]. Moreover, phytoremediation may be subdivided into several processes including: (i) Phytoextraction, the use of plants to remove toxic metals from soil; (ii) phytostabilization, the use of plants to make toxic metal less bioavailable in soil; (iii) rhizofiltration, the use of plants to remove toxic metals from aqueous solution; (iv) rhizodegradation, the use of plant roots to degrade toxic organic contaminants; (v) phytodegradation, the use of plants to take up and degrade toxic organic compounds; (vi) phytotransformation, the plant-assisted transformation of toxic organic compounds into less toxic compounds; and (vii) phytovolatilization, the dispersal of organic compounds taken up into plants and either their dispersal into the air or their partial degradation before being dispersed into the air [7] (Figure 1). Interestingly, these strategies can also be exploited in aquatic environments in order to eliminate dyes, toxic metals, pesticides, hydrocarbons that can represent a risk for both environment and human health [8]–[11].

For phytoremediation to be as effective as possible it is necessary to utilize plants whose growth and development are not inhibited to any significant extent by the presence of the abovementioned toxic compounds. Unfortunately, even plants that are relatively resistant to these toxic substances are likely to grow somewhat more slowly and produce less biomass than plants that are not exposed to these compounds. One way, at least partially, around this problem is to treat the roots (or seeds) of plants being used in phytoremediation protocols with plant growth-promoting bacteria (PGPB). These plant beneficial bacteria have been shown to protect plants against a wide variety of both abiotic and biotic stresses [12]–[19]. In fact, it was previously noted that the addition of PGPB to various plants that were grown in the presence of either metallic or organic environmental contaminants appeared to facilitate the growth of those plants [4],[20]. Here, the more recent use of PGPB to facilitate the phytoremediation of toxic compounds is reviewed and discussed.

## 2. Plant growth-promoting bacteria (PGPB) mechanisms

Most soils worldwide contain very large numbers of microorganisms (millions to billions of microorganisms per gram of soil), both bacteria and fungi [21]. Moreover, these microorganisms typically have a significant effect on the growth and development of plants; soils contain both beneficial PGPB and inhibitory phytopathogenic bacteria, in addition to numerous commensal microbes that have no apparent effect on plant growth [7],[22],[23]. The highest concentrations of soil microbes are generally found on and around plant roots (i.e., the rhizosphere) [24]. The soil distribution of microorganisms is a consequence of the fact that plant roots exude a significant amount of (mostly) small organic molecules which the soil microbes use as a food source [25],[26]. In addition to being located around plant roots, PGPB can also colonize the external surface of aboveground plant tissues i.e., the phyllosphere [27],[28], the spaces between plant cells, especially root cells i.e., the endosphere [29]–[32] or inside of root nodules associated with nitrogen fixation [33],[34].

PGPB encoded mechanisms may facilitate plant growth by (i) interacting directly with a target plant or (ii) indirectly by preventing a phytopathogen (mostly fungi) from inhibiting plant growth and development, in which case the PGPB encoded mechanism(s) often interacts directly with the phytopathogen and not with the plant per se [12],[35]. In addition, most PGPB implement several different mechanisms that are involved in facilitating plant growth [7]. Unfortunately, as a consequence of the multiplicity of ways in which PGPB can promote plant growth, there is no one “super” PGPB strain that can promote plant growth under all environmental conditions. This is because the presence of multiple mechanisms for promoting plant growth in one bacterial strain would likely place a debilitating metabolic load on the proliferation of that bacterium making it difficult for it to survive in the natural environment in competition with other less metabolically encumbered bacterial strains [36]. While there is not one PGPB strain that contains all possible plant growth-promoting mechanisms, researchers have begun to use groups of PGPB or bacterial consortia [18] as a way of more effectively facilitating plant growth especially under stressful conditions such as the presence of environmental contaminants.

**Figure 2.**
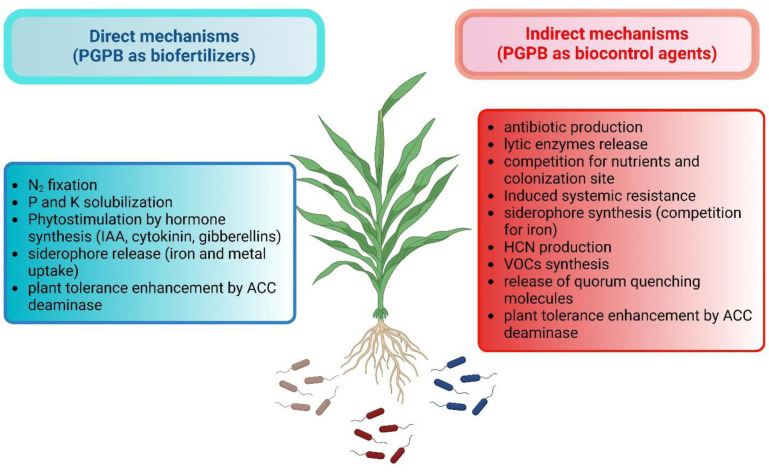
Schematic overview of the main physiological traits of PGPB involved in plant growth promotion including both direct and indirect mechanisms. This figure has been created with BioRender.com.

The most common and best studied direct mechanisms that are used by PGPB include: (i) Solubilizing and facilitating the uptake of minerals from the soil including iron, potassium, and phosphorus; (ii) the fixation of atmospheric nitrogen gas into a soluble form of nitrogen; (iii) the synthesis of plant hormones including cytokinin, gibberellin, and auxin; and (iv) the synthesis of the enzyme 1-aminocyclopropane-1-carboxylate (ACC) deaminase which modulates the plant's level of both ACC and ethylene [7],[12],[37],[38]. The most prominent indirect mechanisms used by PGPB include (i) antibiotic and hydrogen cyanide synthesis; (ii) synthesis of siderophores that can solubilize and sequester iron that prevents the use of iron by nearby phytopathogens; (iii) the ability to synthesize enzymes that are able to digest fungal cell walls; (iv) the ability to outcompete various pathogens for binding to plant roots; (v) the synthesis of volatile organic compounds which can act as inhibitors of some phytopathogen genes; (vi) the synthesis of ACC deaminase to modulate plant ethylene levels and thereby lower plant stress; (vii) the induction of plant systemic resistance; and (viii) the synthesis of quorum quenching molecules [7],[12],[38] (Figure 2).

In addition to the large numbers of many different types of soil bacteria, plant roots are also colonized by plant-beneficial fungi including mycorrhizae [39],[40]. Mycorrhizae have been reported to colonize the roots of more than 90% of all plant species [41]. Mycorrhizae can colonize plant roots either intracellularly or extracellularly. Extracellular colonization of plant roots is achieved by ectomycorrhizae, which bind to the external surface of plant roots. On the other hand, intracellular colonization occurs with endomycorrhizae, also called arbuscular mycorrhiza, where these fungi form unique arbuscules within plant root cells [42]. Plant roots that have been colonized by mycorrhizae exude compounds that act as energy and carbon compound sources for the mycorrhizal fungus thereby enabling the fungus to grow and develop using the plant's resources [43]. Concomitantly, the mycorrhizae that have colonized the plant roots act as a physical extension of the plant's roots and in so doing help provide the plant with minerals and water from a much larger area of the soil than would be possible using only the plant roots, thereby facilitating the plant's growth and development. It has been observed that many PGPB are able to bind and colonize both plant roots and mycorrhizal hyphae; this ability often expedites mycorrhizal colonization of plant roots [44]. The ubiquity of mycorrhizae and their synergism with PGPB is an extremely important adjunct to the growth and development of plants under both stressful and non-stressful conditions.

Employing many of the PGPB encoded mechanisms mentioned above, when these bacteria are used as part of a phytoremediation scheme to remove toxic metals and/or organics from the environment, PGPB can decrease the level of abiotic stress experienced by plants and thereby facilitate the phytoremediation process [7],[20]. The following sections relate, in some detail, recent experiments reporting the use of PGPB to assist the phytoremediation of toxic metals and organic compounds.

## 3. Phytoremediation of metals

The traditional chemical and physical methods used to remediate environments containing toxic metals (such as electro-dialysis, reverse osmosis, extraction, stabilization, soil washing) have been demonstrated to be effective, but are expensive, unsuitable for large contaminated sites, have a high energy requirement and as a consequence of the necessity of using chemical reagents that, together with their toxic waste, could be harmful for the environment, negatively impact on the soil microbiota, and modify soil characteristics [45]–[47].

In recent years, the concept of phytoremediation, which employs the unique capabilities of plants to extract, accumulate, and detoxify contaminants, has emerged as a promising and eco-friendly approach for remediating metal-contaminated soils. However, the efficiency of phytoremediation alone may sometimes be limited by factors such as plant species' tolerance and metal uptake capacity. To overcome these limitations and enhance the efficiency of phytoremediation, researchers have turned their attention to the role of plant-associated bacteria in this process. Beneficial plant-microbe interactions have been shown to significantly contribute to the successful remediation of heavy metal-contaminated soils [48]. Certain bacteria possess unique properties, such as metal chelation, solubilization, and transformation, which aid in the mobilization and bioavailability of metals for plant uptake.

**Figure 3.**
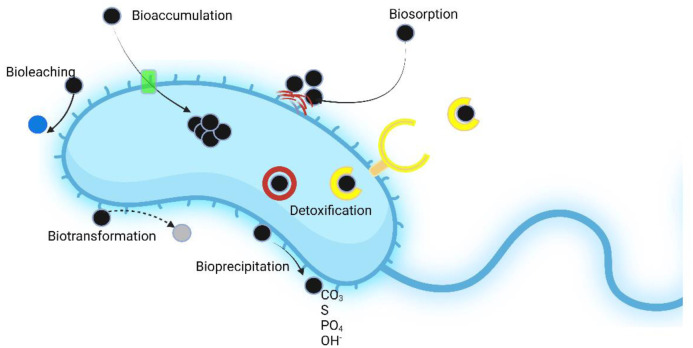
Overview of the main mechanisms used by bacterial cells to cope with toxic metals (black circles). Bioaccumulation is based on the active uptake of the metal (mostly Pb, Ni, Ag, Hg, and Cd) through the membrane via metal transporter proteins (green rectangle) and subsequent concentration inside the cell. Biosorption relies on the capability of the bacterial cells to passively capture metal ions on external cellular surfaces. Bacterial polymers such as exopolysaccharides (EPS; red curved lines) favors metal biosorption, through the establishment of an electrostatic interaction between surface functional groups and the metal ions. Moreover, EPS synthesis leads to biofilm development creating a protective barrier against environmental stresses. Detoxification occurs through the influx of the metal and its sequestration by bacterial cytoplasmic metallothioneins (red circle) or by the release of siderophores (yellow open circle) with a high affinity for iron and other metal ions such as Cd, Cu and Hg. Internalization of the metal-siderophore complex is mediated by specific membrane receptors. Bioprecipitation is based on the binding between the metal and anionic groups located on the cell envelope. Biotransformation involves the chemical transformation of metals (mostly As, Hg, and Cr) into a different molecular form through metabolic reactions such as methylation and demethylation, isomerization, reduction/oxidation. If the product of this reaction is less harmful, the process may also result in detoxification. Bioleaching exploits the ability of some bacterial species (mainly belonging to Acidophiles) to transform the metal in a solid form into a soluble form (blue circle). This bacterial feature may be used to recover metals from ore or metallic alloys. This figure has been created using Biorender™.

In order to consider bacteria as potential agents of assisted phytoremediation it is necessary for the bacteria to be tolerant to the considered pollutant. Metal tolerant PGPB possess a large arsenal of weapons by which they deal with metal pollution, i.e., they can limit metal intake, modify cell permeability, synthesize and release enzymes involved in metal detoxification, bind/immobilize the metal in extracellular or intracellular compartments, actively release metal from the cell and reduce the sensitivity of their own cellular components [49],[50]. The mechanisms used by bacteria interacting with metals are depicted in Figure 3.

As an example, Jain et al. [51] isolated zinc tolerant bacterial strains from a zinc contaminated soil located in the South of Rajasthan, India. According to their level of zinc tolerance (up to 62.5 mM) and plant beneficial activities, four bacterial strains, all belonging to the genus *Serratia*, demonstrated the ability to support the growth of maize plants exposed to zinc contamination. This effect was thought to be related to the IAA, ACC deaminase, and gibberellins synthesized by each of the four isolates as well as to the increased levels of superoxide dismutase, peroxidase, phenylalanine ammonia lyase, catalase and polyphenol oxidase activity detected in inoculated plants. Moreover, the expression of the Zn transporter genes (i.e., ZIP genes) was reduced in plants treated with Zn and inoculated with the bacterial strains.

Metal tolerant PGPB has two main roles in assisted phytoremediation. The first one is favoring plant enrichment of toxic metals through the release of organic acids (formic, tartaric, acetic, succinic, citric, oxalic, and gluconic acids) which improve metal mobility and bioavailability.

The second role is reducing the metal bioavailability by adsorbing them in an exopolysaccharide (EPS) layer or inside biosurfactants that, behaving as metal-complexing agents, lead to metal desorption from solid phases [52]. In the pursuit of effective and sustainable solutions for remediating metal contaminated soils, numerous studies have explored the potential of bacterially assisted phytoremediation; a sample of the most recent papers are provided in Table 1. A computer search (June 2024) on the Web of Science indicates the presence of 1325 papers addressing this topic. Altogether, these studies have sought to unravel the intricate interplay between plants and beneficial bacteria to enhance the efficiency of metal uptake, accumulation, and detoxification.

Table 1 presents a synthesis of some key recent findings from a selection of the last three years of scientific articles on bacterially assisted phytoremediation of metals. Each study investigated distinct aspects of this innovative approach, shedding light on the diverse mechanisms and strategies employed by plant-bacteria partnerships to combat metal pollution. Moreover, an examination of these documents indicates that consortia of two or more bacterial strains with different metabolic features can often result in synergistic positive effects on plant growth as well as on soil remediation.

**Table 1.** A selection of the recent literature on the use of PGPB to ameliorate environmental metal contamination.

Plant species	Inorganic compound	PGPB added	Results	References
*Arabidopsis thaliana* (Thale cress)	Cu	*Cupriavidus metallidurans* CH34	Cu and *C. metallidurans* induced modifications in growth parameters of *A*. *thaliana* such as rosette area, primary and secondary root growth, and dry weight. At Cu level lower than 50 mM an increase in some plant growth parameters was measured, higher concentration induced detrimental effects. A 90% increase and 60% decrease in metal accumulation and mobilization was found in inoculated *A. thaliana* exposed to Cu. Expression of *cop* genes by *C. metallidurans* was upregulated in copper-stressed plants.	[53]
*Basella alba* (Malabar spinach)	Cr (VI)	*Rhodobacter capsulatus* DSM1710	Plants treated with *R. capsulatus* were grown in Cr contaminated soil. Plant growth parameters such as side branching, leaf width, plant dry weights were enhanced by the PGPB. The Cr accumulated by the uninoculated plants was 0.31 mg·kg^−1^, but it increased to 4.02 mg·kg^−1^ in inoculated plants.	[54]
*Centella asiatica* (Indian pennywort)	Pb, Cd	*Enterobacter* sp. FM-1	Strain FM-1 had several PGP traits such as IAA synthesis, production of siderophores, and P-solubilization ability. *C. asiatica* plants inoculated with FM-1 reduced the rhizosphere pH while increasing the bioavailability of both Cd and Pb. Treated plants showed higher Pb and Cd accumulation compared to un-inoculated plants.	[55]
*Chlorophytum comosum* (Spider plant) and *Chlorophytum amaniense* (Mandarin plant)	Cd	*Micrococcus* sp., *Arthrobacter* sp.	The PGPB *Micrococcus* sp. MU1 boosted biomass production of *C. comosum* and C. *amaniense* exposed to Cd. *C. comosum* accumulated a higher level of Cd than *C. amaniense*. Plant inoculation with *Micrococcus* sp. and *Arthrobacter* sp., alone and in combination, enhanced Cd accumulation in shoot and root tissues. The efficiency of Cd phytoextraction by *C. comosum* was higher than that of *C. amaniense*.	[56]
*Festuca arundinacea* (Tall fescue)	Cr	*Bacillus* sp. AK-1 and *Lysinibacillus* sp. AK-5	PGPB inoculation improved plant growth (80.77–139.74% biomass, 60.85–68.04% root length and 7.06–27.10% shoot length). The level of Cr accumulation reached 303.89–372.17 mg/kg in the root, and 16.29–19.29 mg/kg in the shoot.	[57]
*Helianthus annuus* (Sunflower)	Cu	*Pseudomonas lurida* EOO26	Sunflower plants cultivated in Cu polluted soil and inoculated with the PGPB strain showed higher biomass compared to uninoculated plants. The uptake of Cu by treated plants was 8.6- and 1.9-fold greater for roots and leaves than control plants, respectively.	[58]
*Medicago sativa* (Alfalfa)	Cr	*Pseudomonas* sp.	Based on their PGP potential and Cr (VI) tolerance (300–600 mg/L) four strains were used to inoculate *M. sativa* plants; all 4 strains improved plant biomass (shoots and roots). Strain *Pseudomonas* sp. NT27 was most effective in supporting plant growth (97.6 and 95.4% increase of shoot and root dry weights, respectively) and reduced stress symptoms. Inoculated plants showed increased chlorophyll content.	[59]
*Medicago sativa* (Alfalfa)	Cd	*Pseudomonas monteilii* PN1	Cd reduced plant biomass. However, plant inoculation with strain PN1 increased plant biomass and Cd uptake. The levels of auxins, antioxidant enzymes and ACC deaminase were enhanced by inoculation with the PGPB. Although a negative impact of Cd occurred in the rhizosphere microbiota, treatment with strain PN1 partially restored the Cd-based shifts induced in the bacterial community.	[60]
*Miscanthus floridulus* (Giant Japanese silver grass)	Cd	*Klebsiella michiganensis* TS8 and *Lelliottia jeotgali* MR2	*K. michiganensis* TS8 and *L. jeotgali* MR2 were isolated from a Cd contaminated soil for their Cd tolerance and PGP traits. Plant inoculation with the TS8 strain led to increased plant growth parameters (plant height 39.9%, leaf dry weight 99.1%) and reduced Cd concentration in the rhizosphere (–49.2%). Plants inoculated with strain MR2 showed increased metal translocation from the root to the shoot.	[61]
*Pennisetum giganteum* (Giant juncao)	Cd	*Enterobacter cloacae* RCB980, *Klebsiella pneumonia* kpa, *Klebsiella* sp XT-2	*P. giganteum seedlings*, inoculated or not with the three bacterial strains were exposed to increasing Cd levels. Plants treated with PGPB showed a bioaccumulation factor higher than 1.0 and increased values of metal translocation factor compared to uninoculated controls. PGPB consortium boosted the phytoremediation potential.	[62]
*Pteris vittata* (a fern commonly called Chinese brake)	As	*Pseudomonas putida* MK800041, *P. monteilii* MK344656, *P. plecoglossicida* MK345459, *Ochrobactrum intermedium* MK346993, *Agrobacterium tumefaciens* MK346997	*P. monteilii* increased the amount of extractable As (III) and As (V) compared to controls. *P. vittata* plants treated with the PGPB showed greater ability to accumulate As (ranging from 12 to 43%) compared to uninoculated controls. *P. monteilii* was the most efficient PGPB leading to the highest As accumulation (1.9 ± 0.04 g/kg frond) and bioconcentration factor (16.3 ± 0.35) in the fern.	[63]
*Salix integra* (Dappled willow)	Pb	*Bacillus* sp., *Clonostachys rosea* (a fungal endophyte), *Aspergillus niger* (a common mold)	*Bacillus* sp. and *A*. *niger* inoculated in combination increased *S. integra* growth by 14%. The three microorganisms enhanced Pb mobility from soil to the roots (100% compared to uninoculated controls). Single inoculation with either *Bacillus* sp. or *A. niger* increased Pb accumulation inside the plant, mainly aboveground parts. Antioxidant enzymes and proline content were higher in inoculated plants, while soil urease and catalase activities lower in treated plants. 410 metabolites produced by the three microorganisms and belonging to organic acids, amino acids, and carbohydrates were detected, and about half of them modulated heavy metal bioavailability.	[64]
*Sesbania sesban* (perennial legume tree)	Multi metals (Cu, Zn, Cr, Ni)	*Bacillus gibsonii, B. xiamenensis*	*S. sesban* seeds were inoculated with the two bacterial strains (showing plant beneficial traits, and metal tolerance) grown in contaminated and non-contaminated soils. Plant growth was inhibited in multi metal contaminated soil, but plants inoculated with PGPB had longer roots (80–105%) and shoots (75–133%). Pro content and antioxidant enzyme levels were higher in inoculated plants compared to uninoculated ones.	[65]
*Sinapis alba* (White mustard)	Zn	*Pseudomonas fluorescens* MT218317	Zn tolerant *P. fluorescens* MT218317 was used to inoculate *S. alba* plants grown in Zn polluted soil. Inoculated plants showed higher shoot and root growth compared to un-inoculated ones. The bacterium enhanced the amount of Cd and Zn in the plant tissues. The bioaccumulation factors for Cd and Zn were higher in the PGPB treated plants.	[66]
*Zea mays* (Corn)	Cd	*Serratia* sp. CP-13	Cd treatment reduced leaf area, nutrient contents, plant biomass, antioxidant activity, total proteins, photosynthetic pigments especially in a sensitive maize cultivar. Cd also induced an increase of H_2_O_2_, proline, malondialdehyde and relative membrane permeability. Inoculation with PGPB, increased plant biomass, photosynthetic pigments, antioxidative machinery, flavonoids, and proline. Moreover, significant Cd tolerance was acquired by the sensitive maize cultivar.	[67]
*Cannabis sativa* (Hemp) and *Zea mays* (Corn)	As	*Delftia lacustris, Microbacterium oxydans, Microbacterium maritypicum, Bacillus flexus, Delftia tsuruhatensis, Kocuria palustris*	Plant (hemp and corn) inoculation with a consortium of 6 bacterial strains in microcosm and mesocosm conditions increased As accumulation in the aerial parts of the plants by about 10- and 8-fold, in corn and hemp, respectively.	[68]
*Cynodon dactylon* (Bermuda grass), *Eleusine indica* (Indian goosegrass)	Hg	*Jeotgalicoccus huakuii* (B1) and *Bacillus amyloliquefaciens* (B2)	Bermuda grass and Indian goosegrass were planted as monoculture and mixed cropping and inoculated or not with the Hg tolerant bacterial strains *J. huakuii* and *B*. *amyloliquefaciens* alone or in combination. Grass seedlings were cultivated on sand medium spiked with 100 mg/kg HgCl_2_. PGPB inoculation increased grass biomass and Hg bioaccumulation by 52.68% and 47.76%, respectively. The level of Hg in the sand was reduced by 80% by the bacterial consortium.	[69]
*Heliantus annuus* (Sunflower)	Cr (VI)	*Staphylococcus aureus*	Plants were cultivated in Cr polluted soil and treated with cerium dioxide (CeO_2_) nanoparticles and *S. aureus*. CeO_2_ nanoparticles improved both plant development and biomass, alleviated oxidative stress, and increased the enzymatic activities in the plants exposed to Cr stress. Inoculation with *S. aureus* improved the positive effect induced by nanoparticles with the combination nanoparticles + *S. aureus* being the most efficient in phytoremediation.	[70]
*Lolium multiflorum* (European ryegrass)	Pb and Cd	*Pantoea* sp. PP4	Strain PP4 was Pb and Cd tolerant and synthesized auxin, solubilized phosphate. Plants inoculated with PP4 showed higher accumulation of Pb and Cd (28.9% and 95.5%, respectively) compared to un-inoculated controls. Plant fresh and dry biomass, root and shoot length of inoculated *L. multiflorum* increased by 89.2%, 57.1%, 184.6%, and 28.5%, respectively.	[71]
*Melastoma malabathricum* (Malabar melastome)	As	*Microbacterium foliorum*	Plants inoculated with *M. foliorum* showed better phytoremediation efficacy (As tolerance and removal) compared to un-inoculated plants. Treated plants did not display any signs of As toxicity. Both the roots and stems were longer than controls and the bioconcentration factor increased by 0.3 times. *M. foliorum* increased As uptake by the plant both at the root (26%) and at the shoot (22%) level.	[72]
*Oryza sativa* (Rice)	Cd and Zn	*Bacillus* sp. ZC3-2-1	*Bacillus* sp. ZC3-2-1 reduced the amount of bioavailable Cd and Zn in soil (39.3% and 32.0%, respectively), improved the phytoextraction potential in rice (Cd and Zn 48.2% and 8.0%, respectively) and enhanced plant biomass. Strain ZC3-2-1 was also involved in ion homeostasis maintenance through regulation of the Na^+^ and Mg^2+^ concentration in planta and also enhanced Cd–Zn immobilization and improved the enzymatic activities in soil.	[73]
*Phytolacca acinosa*	Cd	*B. cereus* PE31	The endophyte strain *B. cereus* PE31 inoculated on the hyperaccumulator *P. acinosa* enhanced Cd uptake by 42.90% and 28.85% in low and high Cd contaminated soils, respectively. Moreover, the PGPB improved plant growth by increasing plant biomass, as well as Cd concentration and nutrient (N, P, K) availability.	[74]
*Pongamia pinnata* (Pongame oiltree)	Cr (VI)	*Paenibacilus konsidensis* SK3	Inoculation of *P. pinnata* with *Paenibacilus konsidensis* SK3 led to increased Cr (VI) accumulation by 13.45%, 7.56%, and 8.88% in roots, stems, and leaves, respectively.	[75]
*Sedum alfredii* (perennial herb and metal hyperaccumulator)	Multi metals (Zn, Cd, Ni, Cu and Pb)	*Rhodococcus qingshengii*	*R. qingshengii* able to catabolize abscissic acid (ABA) improved the phytoremediation potential of the hyperaccumulator *S. alfredii* towards Zn, Cd, Ni, and Pb compared to uninoculated plants. Inoculation with the bacterial strain increased both the translocation and bioconcentration factor of Zn, Cd, Ni, and Pb in plants. The bioavailability of metals in soil was not affected by the PGPB. *R*. *qingshengii* upregulated the expression of genes (*SaIRT1*, *SaZIP1*, *SaZIP2*, *SaZIP3*, *SaNramp1*, *SaNramp3*, *SaNramp6*, *SaHMA2*, and *SaHMA3*) coding for transporters related to the metal uptake.	[76]
*Sedum alfredii* (perennial herb and metal hyperaccumulator) _ and *Solanum melongena* (eggplant)	Cd	Unidentified strain SaMR12	Eggplant was intercropped with the hyperaccumulator *S. alfredii*. Intercropping induced a detrimental effect on the growth of both plant species, inoculation with the endophyte strain SaMR12 alleviated this growth inhibition. Intercropping treatment boosted Cd accumulation in the edible the part of eggplant, while the inoculation with the PGPB leads to reduction of Cd concentration in eggplant and to an increase in *S. alfredii*. Cd content in eggplant was negatively correlated with the amount of available P and K in the soil, while the Cd concentration in *S. alfredii* is positively correlated with it. The results indicate that the treatment with P and K fertilizers could be beneficial to reduce Cd accumulation in eggplant and improve Cd phytoextraction by *S. alfredii*.	[77]
*Sedum plumbizincicola* (perennial herb)	Cd	*Buttiauxella*, *Pedobacter*, *Aeromonas eucrenophila*, and *Ralstonia pickettii*	Four endophytic bacterial strains inoculated *S. plumbizincicola* grown in a Cd contaminated soil. Following plant inoculation, the pH of the soil was reduced, while the amount of weak acid-extracted Cd and oxidizable Cd increased. The concentration of reducible and residual Cd decreased compared to controls. With a biomass increase of *S. plumbizincicola*, an enhancement of Cd content in plant tissue was detected in inoculated plants. PGPB treatment increased the bioconcentration factor 2.72 times compared to controls. The ability to remove Cd from *S. plumbizincicola* soil was 48.25%. When inoculated, the Cd removal rate of *S. plumbizincicola* reached 71.49%.	[78]
*Solanum nigrum* (Black nightshade)	Cd	*Bacillus paranthracis* NT1 and *Bacillus megaterium* NCT-2	Strains NT1 and NCT-2 inoculated in *S. nigrum* regulate phytohormone synthesis and favour the growth of plants exposed to Cd. Strain NT1 reduced the translocation and bioconcentration factors; strain NCT-2 increased these parameters and improved Cd accumulation in the plant by 41.80%. These PGPB modulated the Cd plant detoxification capability by affecting expression of both antioxidant enzyme *PDR2* genes. Differential expression of metal transport genes *IRT1* and *HMA* in inoculated plants may be responsible for the different level of Cd accumulation measured.	[79]
*Solanum nigrum* (Black nightshade)	Cd	*Bacillus* sp. PGP15	Inoculation of the Cd hyperaccumulator *S. nigrum* with *Bacillus* sp. PGP15 resulted in increased plant biomass and Cd accumulation. Cd-induced plant symptoms were reduced by the PGPB due to decreased H_2_O_2_ contents and oxygen radicals. Superoxide dismutase, ascorbate peroxidase, and catalase activity were higher in the inoculated plants compared to uninoculated ones. Whole genome sequencing of strain PGP15 showed that core genes defining the fundamental metabolic capabilities might not be essential for plant growth promotion.	[80]
*Zea mays* (Corn)	Cr	*Providencia* sp. (TCR05) and *Proteus mirabilis* (TCR20) (this is a human pathogen)	*Providencia* sp. TCR05 and *P*. *mirabilis* TCR20 showed plant beneficial traits, Cr and drought tolerance. Inoculation with the two strains increased maize biomass, pigments, protein, phenolics compound synthesis while it reduced lipid peroxidation, proline, and superoxide dismutase activity under stressful conditions. In presence of metal or/and drought the bacterial strains improved plant photosynthetic activity.	[81]
*Arabis paniculata* (metal hyperaccumulator)	Mn	*Bacillus* sp. AP10	Strain AP10 is a Mn tolerant endophyte able to produce auxins. Strain AP10 increased plant biomass, chlorophyll content and the translocation factor value of Mn in the aerial parts and decreased the malondialdehyde level compared to uninoculated plants. Expression of genes involved in cell-wall loosening, factor involved in plant growth promotion under Mn stress were significantly increased by AP10 treatment. AP10 modulated expression of genes responsible for phenylpropanoid pathway, which may enhance antioxidant flavonoid accumulation involved in reactive oxygen species scavenging to improve tolerance to high level of Mn. AP10 alleviated Mn detrimental effects by boosting the expression of ABA responsive gene.	[82]
*Atriplex lentiformis* (big saltbush)	Cd and Ni	*Bradyrhizobium japonicum* CIM5350 and *Pseudomonas fluorescens* NCIM2100	Plant growth in the presence of two PGPB, organic manure and EDTA was assessed in Cd and Ni contaminated soil. The combination of organic manure with PGPB led to the highest Cd and Ni in plant tissues. Metal uptake by *A. lentiformis* inoculated with strain NCIM2100 was greater than that shown by plants inoculated with NCIM5350. The most efficient metal uptake was detected in plants treated with the two PGPB and organic manure. This treatment also improved plant growth and antioxidant activity. However, EDTA inhibited plant development.	[83]
*Chrysopogon zizanioides* L. (Vetiver)	Cd	*Burkholderia* sp. SRB-1	Treatment with strain SRB-1 promoted growth of *C. zizanioides* and improved the ability to uptake and accumulate Cd in both shoots (36.56%–39.66%) and roots (25.97%–130.47%) compared with un-inoculated control. Plants colonized by SRB-1 showed higher Cd uptake (323.83 mg/kg) than controls (136.28 mg/kg). Strain SRB-1 upregulated expression of *amiE*, *AAO1-2* and *GA2-ox* genes related to auxin and gibberellin biosynthesis. Upregulation of the ATP-binding cassette transporter subfamily genes was noted in *C. zizanioides* roots following PGPB inoculation. In inoculated plants, efficient Cd migration from roots to shoots occurred.	[84]
*Lolium perenne* (perennial ryegrass	Cd	*Bacillus megaterium*	Plant treatment with *B. megaterium* and citric acid boosted the oxidative stress defence and photosynthetic efficiency and increased the rye biomass 1.28 times. This treatment also increased Cd accumulation in the shoot 2.31 times compared to untreated plants. Infrared spectroscopy Fourier Transform associated with a scanning electron microscope Energy Dispersive Spectrometer revealed that the organic constituents such as O-H, C-O and C-N in soils play a key role in Cd mobilization.	[85]
*Lolium perenne* (Perennial ryegrass)	Cd	*Bacillus thuringiensis* SY	Strain SY inoculation enhanced ryegrass root growth and development and increased Cd accumulation in the plants. The bacterial treatment improved plant mineral nutrition and soil enzymatic activities (urease, sucrase, and alkaline phosphatase).	[86]
*Medicago sativa* (Alfalfa)	Multi metals (Pb, Zn, Cu)	*Acinetobacter* L1, *Exiguobacterium acetylicum* L2, and *Klebsiella oxytoca* L3	Under multi metal stress, inoculation of *M. sativa* with immobilized PGPB increased plant biomass (19.8, 6.89, and 14.6%, root, stem and leaf dry weight, respectively). Also, the plant antioxidant capability was improved by treatment with immobilized bacterial strains.	[87]
*Miscanthus floridulus* (Pacific Island silvergrass)	Cd	*Bacillus cereus* BL4	Plants inoculated with Cd-tolerant *B. cereus* BL4 showed increased biomass, height, and Cd accumulation in shoots (32.26%, 16.60%, and 24.88%, respectively) and roots (56.41%, 33.93%, and 42.37%, respectively). Plant inoculation led to enhanced chlorophyll content, photosynthetic rate, and root activity under Cd stress. The glutathione content as well as antioxidant enzymes increased after BL4 treatment. The membrane permeability and malonaldehyde concentration was reduced by inoculation with BL4, suggesting an alleviation of Cd cytotoxicity. The availability of the metal and soil enzymatic activities were enhanced by BL4.	[88]
*Miscanthus giganteus* (Giant perennial grass)	Zn	*Mycolicibacterium* sp. Pb113 and *Chitinophaga* sp. Zn19	Plants were inoculated with strain Pb113 and strain Zn19 and grown in Zn contaminated soil (1650 mg/kg). The rhizosphere microbiota was modified by the metal more than by the inoculant. Both inoculants supported plant development while only strain Zn19 enhanced Zn accumulation in plant tissues, especially at the shoot level.	[89]
*Spinacia oleracea* (Spinach)	Multi metals (Cd, Ni, Cu, Zn, Mn)	*Bacillus* sp. TC4 *Bacillus circulans* TC7 *Pseudomonas* sp. TC33 *Bacillus subtilis* TC34 *Terribacillus* sp. TC45	Multi metal contamination inhibits plant growth measured as root and shoot length (42.8% and 60.1%, respectively), biomass (80%), chlorophyll content (41%), soil phosphatases (42%), and urease (42%) activity. PGPB inoculation alleviated plant stress and increased spinach growth (74.5% for root length, 106.3% for shoot length, and up to 5.5-fold for fresh weight) and enhanced soil enzymatic activities. While *B. circulans* TC7 favoured plant growth, it reduced metal accumulation. On the contrary, *Pseudomonas* sp. TC33 and *B. subtilis* TC34 increased metal accumulation.	[90]
*Suaeda salsa* (Seepweed)	Cd	*Escherichia coli* 10527	Strain 10527 is P solubilizing and can colonize the halophyte *S. salsa*. The Cd extraction efficiency of the plant was improved by the bacterial strain; this effect has been related to the remodelling of the rhizosphere microbiota. Strain 10527 strengthened the interactive effects of keystone taxa in the rhizosphere soil and enriched the numbers of bacterial species considered as key drivers in Cd mobilization.	[91]
*Vigna radiata* (Mung bean)	Cr	*Bacillus, Microbacterium* and *Pseudomonas* spp.	Seedlings were inoculated with a mixture of phosphate solubilizing bacteria and PGPB and grown in a Cr contaminated soil. While Cr induced detrimental effects on the host plant (–44%, –72%, –68%, and –49% in root and shoot length, leaf number and leaf area, respectively) compared to un-inoculated controls, inoculation with the bacterial consortium alleviated Cr toxicity and improved plant development and chlorophyll.	[92]
*Zea mays* (Corn)	As, Hg	*Pseudomonas fluorescens* UM270 and *Bacillus paralicheniformis* ZAP17	Plants exposed to As and Hg stress were inoculated with a mixture of *P. fluorescens* UM270 and *B. paralicheniformis* ZAP17. Treatment with HAsNa_2_O_4_ in the presence of the two bacterial strains promoted shoot length and plant biomass. Plants treated with HgCl_2_ and inoculated with the consortium showed higher plant biomass, and root and shoot length compared to control plants.	[93]

## 4. Phytoremediation of organic contaminants

**Figure 4.**
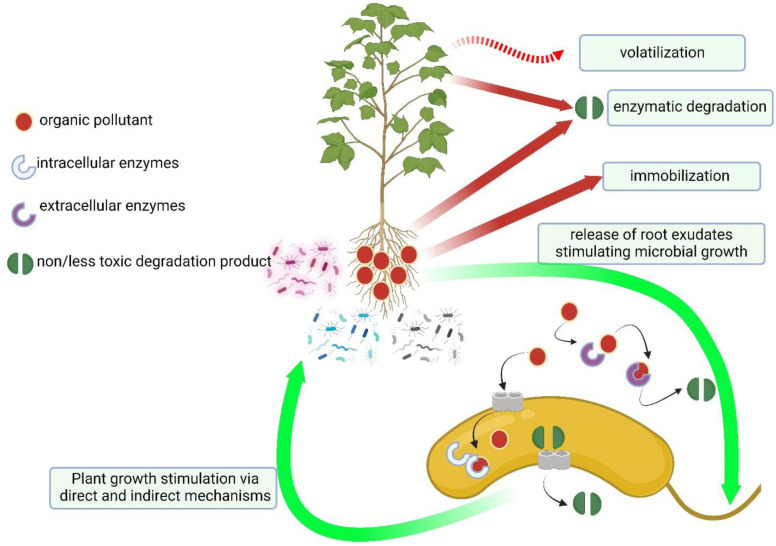
Assisted phytoremediation of organic pollutants: Plants can immobilize organic pollutants in the roots, volatilize them from the leaves or the shoot, or metabolize them both in the root and in the shoot through transformation (redox or hydrolysis reactions), conjugation with molecules in order to reduce the toxicity, and compartmentalization in the vacuole or in cell wall. Moreover, organic pollutants can be degraded by plant-associated bacteria living in the rhizosphere via intra- and extra-cellular enzymes, thus reducing their phytotoxicity. While plants support the growth of root associated bacteria by the rhizodeposition, bacteria behaving as PGPB promote plant growth via direct and indirect mechanisms. This figure was created with BioRender.com.

Due to their recalcitrant nature and potential toxicity, organic contaminants, especially polychlorinated biphenyls (PCBs), polycyclic aromatic hydrocarbons (PAHs), and chlorinated solvents, exert adverse effects on ecosystems and public health [94]–[98]. Traditional remediation techniques are frequently found to be inadequate in addressing the complex nature of these pollutants, thus highlighting the requirement for innovative approaches for their effective remediation. In recent years, phytoremediation, based on the promising capabilities of plants to absorb, degrade, or sequester contaminants, has gained increasing attention as an ecologically sound solution to combat organic pollutant contamination. Phytoremediation of organic pollutants in soil occurs through degradation or transformation of the pollutant molecule. In phytodegradation the pollutant is adsorbed with different strategies according to its physical and chemical characteristics (i.e., hydrophobic/hydrophilic properties) and metabolized or degraded into less harmful molecules and distributed within plant tissues or volatilized into the atmosphere (Figure 4). If the degradation of the organic pollutant occurs in the rhizosphere the process is identified as rhizodegradation [99].

The major advantages of this approach are low cost, aesthetic appeal, and minimal environmental disruption. These characteristics make it an attractive and sustainable option for environmental cleanup. However, when applied to organic pollutants phytoremediation often shows several limitations including relatively slow remediation rates, species-specific uptake capabilities, and challenges associated with contaminant mobility in the environment [100]. These factors can hamper the effectiveness of phytoremediation, particularly in scenarios where fast and efficient cleanup is required. To address these limitations and enhance the efficiency of phytoremediation, the use of plant-associated bacteria can play a pivotal role in accelerating the degradation and detoxification of organic pollutants in the root zone of plants. This collaborative remediation strategy offers several distinct advantages: 1) Quick remediation: Bacteria can boost and speed up the clean-up ability of the host plant; 2) wide spectrum remediation: Plant-associated bacteria can expand the range of organic pollutants that can be effectively remediated, including some otherwise recalcitrant and complex molecules; 3) high adaptability: Following contamination, the bacterial community structure can shift in its composition and quickly adapt to the new condition thus ensuring the continued efficacy of the remediation process. In addition, in the absence of the appropriate bacterial strains, several organic pollutants may be toxic for the plants used in phytoremediation. In this context, many PGPB can reduce the harmful effects of the toxic organic molecules through 1) amelioration of plant nutritional requirements (phosphate solubilization, nitrogen fixation, iron uptake by siderophores), 2) plant health improvement (behaving as a biocontrol agent against phytopathogenic organisms), 3) modulation of the phytohormone pathways and 4) plant tolerance enhancement via ACC deaminase synthesis thereby lowering plant stress ethylene levels, and 5) degradation of the contaminant into less toxic molecules [101] (Figures 1 and 4). Obviously, the efficacy of different PGPB in supporting phytoremediation varies according to a multitude of parameters such as the soil nutrient content, the interactions among soil microorganisms and those occurring between microorganisms and other biota, possible horizontal gene transfer, availability of the pollutant, and the oxygen concentration. The efficiency of bacterial degradation also depends on the complexity of the pollutant molecule. As an example, focusing on petroleum hydrocarbons, molecules with a simple chemical structure (i.e., alkanes) can be rapidly degraded, while those with a complex structure, such as polycyclic aromatic hydrocarbons, are more difficult to degrade [102]. However, the combination of the plant with the bacterial inoculant can often make a significant difference. As an example, in a very recent paper, Guan et al. (2023) [103] described phenanthrene removal using *Brassica napus* and the PGPB *Serratia* sp. DLN5. Canola was cultivated in a soil contaminated with 100 mg·kg^−1^ phenanthrene and natural degradation by the native soil microorganisms lead to a reduction of the soil phenanthrene concentration to 60.8 mg·kg^−1^. However, the presence of the plant and the added PGPB improved the removal efficiency of the pollutant by 71.5%–82.5%. Furthermore, the *Serratia* sp. strain increased the plant biomass, enhanced the root length (+22.2%) and the net photosynthetic rate (+334.9%). Finally, plant inoculation with the PGPB induced shifts in the plant associated microbiota stimulating the proliferation of bacterial species involved in phenanthrene degradation such as *Flavobacterium*, *Methylophilaceae*, and *Burkholderiaceae*.

Importantly, the role of endophyte bacteria colonizing plant internal tissues without damaging the plant but facilitating the remediation of organic pollutants should be mentioned. These bacteria benefit from intimate contact with the plant cells, interacting more efficiently with their host and living in a protected niche where a sufficient level of nutrients is available. Interestingly, several papers emphasized the fact that endophytic strains performed better than rhizospheric ones in the degradation of organic pollutants. In fact, detailed molecular studies have revealed that endophytes have a greater number of genes involved in contaminant removal compared to bacteria merely colonizing the rhizosphere. Apparently, this is also related to a high rate of horizontal gene transfer to native endophytes or to the host followed by gene duplication [104]–[107].

The clear result from a large number of publications (Table 2) is that clean up technologies based on the exploitation of the symbiotic relationship between plants and PGPB represent a significant opportunity when approaching an organic pollutant remediation plan.

**Table 2.** A selection of the recent literature on the use of PGPB to ameliorate environmental organic contamination.

Plant species	Organic compound	PGPB added	Results	References
*Festuca* spp. (Fescue) and *Echinacea purpurea* (Purple coneflower)	Polycyclic aromatic hydrocarbon (PAH) and Cd	*Mycobacterium* N12	*Mycobacterium* N12 was used as inoculant of *Festuca* L. and *Echinacea purpurea* grown in soil contaminated with PAH and Cd. While plant treatment did not improve the pollutant remediation efficiency of *E*. *purpurea*, inoculation with the PGPB improved *Festuca* development and its capability to remove both PAH (+76.3%) and Cd (+68.3%) compared to uninoculated controls. After 150 days the root biomass of inoculated plants increased by 59.40% compared to uninoculated ones.	[108]
*Melia azedarach* (Chinaberry)	benzo(a) pyrene	*Bacillus subtilis* SR1	The genome of *B. subtilis* SR1, tolerant to metals and able to degrade PAH consists of a circular chromosome (4,093,698 bp) including 4155 genes and 4095 proteins. Several catabolic genes, such as aromatic ring-hydroxylating dioxygenase, aromatic ring hydroxylase, catechol 2,3 dioxygenase, 4-hydroxybenzoate decarboxylase, and carboxymuconolactone decarboxylase involved in the aromatic hydrocarbon degradation were found together with genes encoding biosurfactants and genes determining tolerance to Cd, Zn and Co. The bacterial strain catabolized up to 35% benzo(a) pyrene after 21 days of *in-vitro* growth. When inoculated in *M. azedarach* degradation rate reached 85%.	[109]
*Pteris vittata* (an Asian fern)	As and phenanthrene	*Alcaligenes* sp.	Inoculation of the As hyperaccumulator *P. vittate* with *Alcaligenes* sp. enhanced the breakdown of phenanthrene in soil. Moreover, modifications of As bioavailability occurred through increase of soil pH, solubilization of minerals such as Fe and Ca, and faster organic matter decomposition. Significant variability was observed between plant genotypes in phenanthrene breakdown.	[110]
*Zea mays* (Corn)	Petroleum hydrocarbons	*Bacillus* sp. MN54	The impact of sugarcane bagasse biochar with *Bacillus* sp. MN54 on phytoremediation of petroleum and on maize development in a diesel contaminated soil was assessed. Seeds of *Z. mays* treated with biochar and *Bacillus* sp. MN54 were sown in uncontaminated or contaminated soil substrates. Petroleum hydrocarbons inhibited plant growth while inoculation with the strain MN54 and biochar significantly reduced phytotoxicity and improved N, P, and K uptake by 41%, 43% and 37%, respectively. Moreover, the combined treatment led to 77% removal of the pollutant.	[111]
*Lolium multiflorum* (Ryegrass)	Total petroleum hydrocarbons (TPHs)	*Acinetobacter bouvetii*, *Pseudomonas rhizosphaerae* and *Stenotrophomonas rhizophila* + biochar + compost	Remediation of a TPH contaminated soil was realized using Italian ryegrass, treated with compost, biochar and a consortium of bacterial strains immobilized in biochar. The highest rate of TPH removal (40%) was induced by the mixed treatment consisting of compost + biochar + immobilized bacteria, compared to other treatments. Plants treated with the same inoculum showed the higher root biomass (+85–159%).	[112]
*Azadirchta indica* (Mahogany family tree) or *Delonix regia* (Royal poinciana)	Crude oil	*Gordonia amicalis* BB-DAC, *Pseudomonas aeruginosa* BB-BE3, *P. citronellolis* BB-NA1, *Rhodococcus ruber* BB-VND, and *Ochrobactrum anthropi* BB-NM2.	Five bacterial strains (*Gordonia amicalis* BB-DAC, *Pseudomonas aeruginosa* BB-BE3, *P. citronellolis* BB-NA1, *Rhodococcus ruber* BB-VND, and *Ochrobactrum anthropi* BB-NM2) able to degrade hydrocarbons were isolated from the rhizosphere of native plants growing in a crude oil polluted site A bacterial consortium was used to inoculate *Azadirchta indica* or *Delonix regia* cultivated in open field conditions in a crude oil-contaminated site. Shifts in the rhizosphere microbiota consisted of enhancement of bacterial species able to breakdown the pollutant. After 120 days of plant cultivation degradation of petroleum hydrocarbons reached 67% with *A. indica* and 55% with *D. regia* with the same treatment.	[113]
*Brassica napus* (Canola)	Phenanthrene	*Serratia* sp. DLN5	A PGPB able to degrade phenanthrene was isolated and identified as *Serratia* sp. strain DLN5. Once inoculated in canola grown in phenanthrene contaminated soil (200 mg·kg^−1^), the PGPB improved plant biomass, increased root length (+22.2%) and the net photosynthetic rate (+334.9%) while reducing the amount of malonaldehyde in roots. Moreover, plant inoculation with strain DLN5 modified bacterial community structure stimulating bacterial species involved in phenanthrene degradation.	[103]
*Cannabis sativa* (Cannabis)	Multi metals and Diacetone alcohol, Pentasiloxane, Erythritol, 2,6-Bis(tert-butyl)phenol, 3-Chloropropionic acid, 2-Pyrrolidinone, Cyclic octaatomic sulfur, Cyclohexane, 1,3,5-Benzetriol, Lupan-3-ol, acetate, 2,4-Dihydroxybenzoic acid, 1-Octacosanol, 24-Ethyl-ë(22)-coprostenol, á-Sitosterol, Tris(2,4-di-tert-butylphenyl) phosphate	*Bacillus thuringiensis* MW887525, *Bacillus* cereus MW887524, *Achromobacter denitrificans* MW886333, *Bacillus subtilis* MW886231	Four out of seven bacterial strains isolated from the rhizosphere sludge of *C. sativa* were selected and identified as *B. thuringiensis* MW887525, *B. cereus* MW887524, *A. denitrificans* MW886333 and B. subtilis MW886231. These strains, showing PGP features and ligninolytic activity, were inoculated onto *C. sativa* growing on fresh disposed distillery sludge. After 30 and 60 days the conversion and disappearance of organic compounds occurred through by the activity of the microorganisms. Moreover, inoculated plants showed a higher Fe, Cu, Zn, Mn, Pb, Ni, Cd and Cr accumulation into the plant tissues.	[114]
*Festuca arundinacea* (tall fescue)	Phenanthrene and pyrene	*P. fluorescens* Ps2-6 + AM fungus (G. versiforme)	Tall fescue inoculation with the consortium by *G. versiforme* and the *P. fluorescens* improved plant growth and increased the phenanthrene and pyrene accumulation in plants. Moreover, the combined treatment induced shifts in microbial community of the polluted soil characterized by a boost in *Proteobacteria* (*Sphingomonas*, *Pseudomonas*, and *Fusarium* genera).	[115]
*Juncus effusus* (perennial herbaceous rush)	Pyrene and nickel	*Klebsiella pneumoniae*	*J. effusus* plants were inoculated with *K. pneumoniae* and grown in pots in soil polluted with nickel and pyrene. The bacterial strain increased plant biomass and nutrient uptake and enhanced the pyrene degradation rate in soil (97%). Similarly, the amount of Ni accumulated into the roots increased following bacterial inoculation. The PGPB alleviated the stress induced on the plant by the presence of pyrene and nickel.	[116]
*Medicago sativa* (Alfalfa)	Crude oil	*Bacillus subtilis* PM32Y, *Bacillus cereus* WZ3S1, *Bacillus* sp. SM73 and *Bacillus* sp. WZ3S3	Eight bacterial strains isolated from a petroleum polluted soil were used as inoculant in alfalfa grown in sand contaminated with 10 g crude oil per kg. After 60 days of growth, plants inoculated with *Bacillus subtilis* PM32Y, *Bacillus cereus* WZ3S1, *Bacillus* sp. SM73 and *Bacillus* sp. WZ3S3, all able to synthesize ACC deaminase, degraded petroleum hydrocarbons. *B*. *subtilis* PM32Y was the most efficient strain in stimulating plant degradation of the organic pollutant (up to 47%).	[117]
*Opuntia ficus*-*indica* (Barbary fig), *Eucalyptus camaldulensis*, (River red gum) and Nerium oleander (Nerium)	Total petroleum hydrocarbons (TPH)	*Bacillus pumilus*, *Pseudomonas putida*	The three plant species were inoculated with *B. pumilus* and *P. putida*, alone or in combination. Degradation of TPH was highest in *E. camaldulensis* inoculated with the consortium, removing 77.30% of soil TPH. Inoculation of P. putida in *E. camaldulensis* reduced TPH by 69.60%. In general, *P. putida* was more efficient than *B. pumilus* in TPH removal.	[118]
*Suaeda salsa* (Seepweed)	Phenanthrene	*Martelella* sp. AD-3	Bacterial strain *Martelella* sp. AD-3 was immobilized in biochar and used to inoculate *S. salsa* growing in saline-alkali soil. The phenanthrene removal rate after 40-days of plant growth reached 91.67 %. Plant biomass and leaf pigment concentration increased 1.30 and 1.35 times, respectively. Soil treatment with the plant and associated bacteria induced shifts in the microbial community and an increase frequency of bacteria able to degrade PAH.	[119]
*Triticum aestivum* (Wheat)	Total petroleum hydrocarbons	*Sphingobacterium spiritivorum* MH-9 + *Alcaligenes faecalis* MH-2 *Sphingobacterium spiritivorum* MH-10 + *Stenotrophomonas rhizophila* MH-24	Several bacterial strains were isolated from a petroleum hydrocarbon polluted soil. Two kinds of consortia were developed and used as inoculants of *T. aestivum* grown in pots with soil contaminated with petroleum hydrocarbons. The organics strongly inhibit plant development (–18–37% and –14–34% in agronomic and physiological plant parameters, respectively). Inoculation with the bacterial consortia led to increases of both agronomic and physiological plant parameters (+32% and +27%) compared to un-inoculated controls. Moreover, plant treatment improved nutrient uptake and activated antioxidant pathways. While wheat remediated 48% of the initial concentration of petroleum hydrocarbons, inoculation with the two consortia boosted the remediation rate to 78%.	[120]
*Vigna unguiculata* (Black-eyed pea)	Tapis crude oil	*Microcococcus luteus* WN01	A rhizobox was divided into three compartments i.e., the rhizosphere, the mid-zone, and the bulk soil zones. *V. unguiculata* was cultivated in this system for 1 month and the roots growing in the rhizosphere compartment were inoculated with *M. luteus* WN01. Plant treatment with *M. luteus* WN01 increased cowpea root biomass as well as the release of root exudates. Moreover, the highest removal efficiency, microbial activities, microbial density, and bacterial biodiversity were observed in the rhizosphere compartment.	[121]
*Zea mays* (Corn)	Total petrol hydrocarbons	Consortium 1: *Dietzia* sp. IN118, *Gordonia* sp. IN101, *Mycolicibacterium frederiksbergense* IN53, *Rhodococcus erythropolis* IN119, *Rhodococcus globerulus* IN113, *Raoultella* sp. IN109 Consortium 2: consortium 1+ *Aspergillus sydowii*, *Aspergillus versicolor*, *Candida* sp., *Cladosporium halotolerans*, *Penicillium chrysogenum*	Inoculation of soil contaminated with petrol hydrocarbons with consortium 1 led to reductions of TPH and PAH by 31.85% and 27.41%, respectively. Consortium 2 was more effective than consortium 1 and was able to decrease TPH by 41.67% and PAH by 34.73%. When consortium 2 was used to inoculate Z. mays the reduction of TPH and PAH reached 65.35% and 60.80%, respectively. Toxicological assays supported the remediation efficiency of consortium 2 associated with *Z. mays* plants.	[122]

## 5. Summary and Conclusion

As human civilizations have grown and developed, they have created an enormous amount of waste material, much of which has been dumped into the natural environment, which until relatively recently was considered to be an almost infinite sink for these discarded materials. Unfortunately, much of this discarded material, in addition to being unsightly and ruining the natural environment, is potentially toxic to microbial, plant, animal, and human life. While the ultimate solution to this problem is to prevent the use and wanton disposal of toxic metals and organic compounds, a solution which may take many years to implement to any significant extent, it is essential that in the meantime we remediate the hundreds of thousands of waste sites of toxic metals and organic compounds that currently exist all over the globe.

Here, a selection of the many hundreds of studies that have very recently been undertaken by scientists in an effort to inexpensively remediate toxic waste sites containing toxic metals and organic compounds using plants (i.e., phytoremediation) is addressed. In particular, all of the studies summarized in sections 3 and 4 (above) include the presence of added microorganisms that facilitate plant growth (i.e., PGPB) and the uptake or breakdown of either metallic and/or organic toxicants. These plant-microbe phytoremediation partnerships have been successfully studied in a variety of settings from the laboratory to the greenhouse to the field, and it is now clear that an approach to phytoremediation using a combination of plants and microbes is the not only the least expensive way to clean up the environment, but it is also the most efficacious. Perhaps, the main drawback of this approach is that it typically requires several seasons of plant growth to remove all or most of the toxic chemicals from the field.A number of factors separate these recent studies from earlier studies of PGPB-assisted phytoremediation. (i) Instead of employing agricultural plants to remove soil contaminants, for the most part, the very recent studies have employed a wide range of non-agricultural plants including a variety of grasses that grow to a relatively large size. (ii) Using a range of mechanisms to promote plant growth, many newly isolated and characterized PGPB significantly improve the phytoremediation ability of most plants. (iii) Both rhizosphere and endophyte PGPB strains have been tested as components of various phytoremediation schemes and both types of bacteria have been found to be highly effective. (iv) While individual PGPB strains are often effective in assisting phytoremediation (including plant growth and development in the presence of environmental contaminants), consortia of several PGPB are often more effective than individual PGPB. (v) A few of the reports describing highly effective bacterial facilitation of phytoremediation fail to note that the bacteria used in those studies are potential human pathogens. While it may be acceptable to use these bacterial strains in laboratory experiments, notwithstanding their effectiveness in the laboratory, it is not acceptable to utilize these bacteria in the field.

## Use of AI tools declaration

The authors declare they have not used Artificial Intelligence (AI) tools in the creation of this article.
